# Inhibition of Brain Swelling after Ischemia-Reperfusion by *β*-Adrenergic Antagonists: Correlation with Increased K^+^ and Decreased Ca^2+^ Concentrations in Extracellular Fluid

**DOI:** 10.1155/2014/873590

**Published:** 2014-11-13

**Authors:** Dan Song, Junnan Xu, Ting Du, Enzhi Yan, Leif Hertz, Wolfgang Walz, Liang Peng

**Affiliations:** ^1^Laboratory of Brain Metabolic Diseases, Institute of Metabolic Disease Research and Drug Development China Medical University, No. 92 Beier Road, Heping District, Shenyang 110001, China; ^2^Departments of Psychiatry and Pharmacology, University of Saskatchewan, Saskatoon, SK, Canada

## Abstract

Infarct size and brain edema following ischemia/reperfusion are reduced by inhibitors of the Na^+^, K^+^, 2Cl^−^, and water cotransporter NKCC1 and by *β*_1_-adrenoceptor antagonists. NKCC1 is a secondary active transporter, mainly localized in astrocytes, driven by transmembrane Na^+^/K^+^ gradients generated by the Na^+^,K^+^-ATPase. The astrocytic Na^+^,K^+^-ATPase is stimulated by small increases in extracellular K^+^ concentration and by the *β*-adrenergic agonist isoproterenol. Larger K^+^ increases, as occurring during ischemia, also stimulate NKCC1, creating cell swelling. This study showed no edema after 3 hr medial cerebral artery occlusion but pronounced edema after 8 hr reperfusion. The edema was abolished by inhibitors of specifically *β*_1_-adrenergic pathways, indicating failure of K^+^-mediated, but not *β*_1_-adrenoceptor-mediated, stimulation of Na^+^,K^+^-ATPase/NKCC1 transport during reoxygenation. Ninety percent reduction of extracellular Ca^2+^ concentration occurs in ischemia. Ca^2+^ omission abolished K^+^ uptake in normoxic cultures of astrocytes after addition of 5 mM KCl. A large decrease in ouabain potency on K^+^ uptake in cultured astrocytes was also demonstrated in Ca^2+^-depleted media, and endogenous ouabains are needed for astrocytic K^+^ uptake. Thus, among the ionic changes induced by ischemia, the decrease in extracellular Ca^2+^ causes failure of the high-K^+^-stimulated Na^+^,K^+^-ATPase/NKCC1 ion/water uptake, making *β*_1_-adrenergic activation the only stimulus and its inhibition effective against edema.

## 1. Introduction

Cellular edema is a dreaded consequence of ischemic stroke. On account of the fixed volume of the skull changes in cellular and extracellular volume can compromise cerebral blood flow and metabolism or result in compression of vital brain structures. Novel potential treatment targets for cerebral edema include the Na^+^-K^+^-2Cl^−^ cotransporter NKCC1, a critical mediator of edema formation [1]. Its specific inhibitor, bumetanide, inhibits ischemia-reperfusion-induced edema in experimental stroke after middle cerebral occlusion (MCAO) followed by reperfusion [2–4], and knock-out of NKCC1 reduces brain swelling by one-half following 2 hr of MCAO and 24 hr of reperfusion [5].

Dysregulation of ion homeostasis plays a major role in the formation of brain edema. Brain slices show pronounced swelling (edema) during exposure to highly elevated K^+^ concentrations [6, 7] and the swelling increases with the extracellular K^+^ concentration up to ~50 mM [8]. Pronounced increases in extracellular K^+^ concentrations rapidly occur during brain ischemia together with a massive decrease in extracellular Ca^2+^ and a more moderate decrease in extracellular Na^+^ [9]. In adult brain NKCC1 is mainly expressed in astrocytes [10–13]. They are also the cells that show most swelling after exposure to pathologically elevated extracellular K^+^ concentration [14–16].

NKCC1 is not an active transporter but its operation is driven by ion gradients established by Na^+^,K^+^-ATPase activity, making it a secondary active transporter [18–20], which requires ongoing or preceding Na^+^,K^+^-ATPase activity in order to function. In turn, function of the Na^+^,K^+^-ATPase is critically dependent upon the presence of oxygen and a metabolic substrate, generally glucose. The astrocytic Na^+^,K^+^-ATPase is normally stimulated by slightly elevated extracellular K^+^ concentrations [21–23]. The resulting stimulation of K^+^ uptake requires K^+^-activated signaling by endogenous ouabains at low nM concentrations, which includes an increase in free cytosolic Ca^2+^ concentration ([Ca^2+^]_i_) evoked by activation of the IP_3_ receptor [17]. This leads to an uptake of Na^+^, which is required for concomitant stimulation of the intracellular Na^+^-sensitive site of the astrocytic Na^+^,K^+^-ATPase and compensates for the absence of astrocytic Na^+^ entry during neuronal excitation. In neurons the effect of low nanomolar ouabain concentrations on [Ca^2+^]_i_ is known to be abolished by a blocker of the Na^+^-Ca^2+^ exchanger (NCX) [24]. The same authors also showed ouabain-mediated Ca^2+^ release from the endoplasmic reticulum in astrocytes but unfortunately no information was provided about potential effects of NCX. This would have been important, because inhibition of astrocytic ouabain signaling and thus Na^+^,K^+^-ATPase function by deficient Ca^2+^ entry after ischemia/reperfusion-induced depletion of extracellular Ca^2+^ [9] might inactivate the normally occurring K^+^-induced stimulation of Na^+^,K^+^-ATPase activity required for NKCC1 function [17]. This would make NKCC1 stimulation dependent upon *β*-adrenergic signaling, which also stimulates astrocytic, but not neuronal, Na^+^,K^+^-ATPase activity [23]. It would mean that following ischemia/reperfusion NKCC1 activity would be selectively dependent upon *β*-adrenergic stimulation. Such dependency might explain the protective effect of *β*-adrenergic antagonists demonstrated in animal models of CNS ischemia [25–28].

In the present study the increase in brain edema by unilateral MCAO and its abolishment by an NKCC1 inhibitor and a *β*_1_-adrenergic antagonist were confirmed. Moreover, whether the increase occurred during the ischemic period or was delayed until after reoxygenation and whether inhibition of the known pathway for *β*_1_-adrenergic stimulation of cultured astrocytes also prevented edema formation were tested. The inhibitory effect of extracellular Ca^2+^ deficiency on K^+^ uptake was tested in two different types of experiments. One of these compared effects of a slightly elevated extracellular K^+^ concentration and of the *β*_1_-adrenergic agonist dobutamine on uptake of K^+^ into primary cultures of astrocytes during incubation in a medium with a normal Ca^2+^ concentration and in a Ca^2+^-depleted medium. The other showed that the inhibitory effect of *μ*M concentrations of ouabain on K^+^ uptake in cultured astrocytes is much less potent in the absence of Ca^2+^ in the incubation medium.

## 2. Methods

### 2.1. Materials

Most chemicals, including isoproterenol, ethacrynic acid, ouabain hydrochloride, the *β*-adrenergic receptor antagonists, betaxolol (4-(2-cyclopropylmethoxyethyl)-1-phenoxy-3-isopropylaminopropan-2-ol) and ICI118551 (erythro-(±)-1(7-methylindan-4-yloxy)-3-isopropylaminobutan-2-ol), the *β*_1_-adrenergic agonist dobutamine, pertussis toxin (PTX), and the PKA inhibitor H-89 (N-[2-(p-bromocinnamylamino)ethyl]-5-isoquinolinesulphonamide) were purchased from Sigma (St. Louis, MO, USA). The EGFR tyrosine kinase inhibitor, Tyrphostin AG 1478 (N-[(2R)-2-(hydroxamidocarbonymethyl)-4-methylpentanoyl]-L-tryptophan methylamide); the metalloproteinase inhibitor, GM 6001 (1,4-diamino-2,3-dicyano-1,4-bis[2-aminophenylthio]butadiene); the MEK inhibitor, U0126, (1,4-diamino-2,3-dicyano-1,4-bis[2-aminophenylthio]butadiene); and the Src inhibitor, PP1 (4-amino-5-(4-methylphenyl)-7-(t-butyl)pyrazolo[3,4-d]pyrimidine) were obtained from Calbiochem (La Jolla, CA, USA). Horse serum was either drawn from selected animals (Figure 3) or bought from Gibco (Gaithersburg, MD, USA). The surfactant polyol Pluronic F-127 and P-1267 PBFI-AM (124549-23-1/1,3-benzenedicarboxylic acid, 4,4′-[1,4,10,13-tetraoxa-7,16-diazacyclooctadecane-7,16-diylbis(5-methoxy-6,2-benzofurandiyl)]bis-, tetrakis[(acetyloxy)methyl]ester) used for fluorescence determination of intracellular K^+^ concentration were purchased from Invitrogen (Carlsbad, CA, USA). ^42^K was obtained from New England Nuclear, Lachine, Quebec (no longer available). Pentobarbital sodium was obtained from Sinopharm Chemical Reagent Co., Ltd. (Shanghai, China).

### 2.2. MCAO

**Figure 1:**
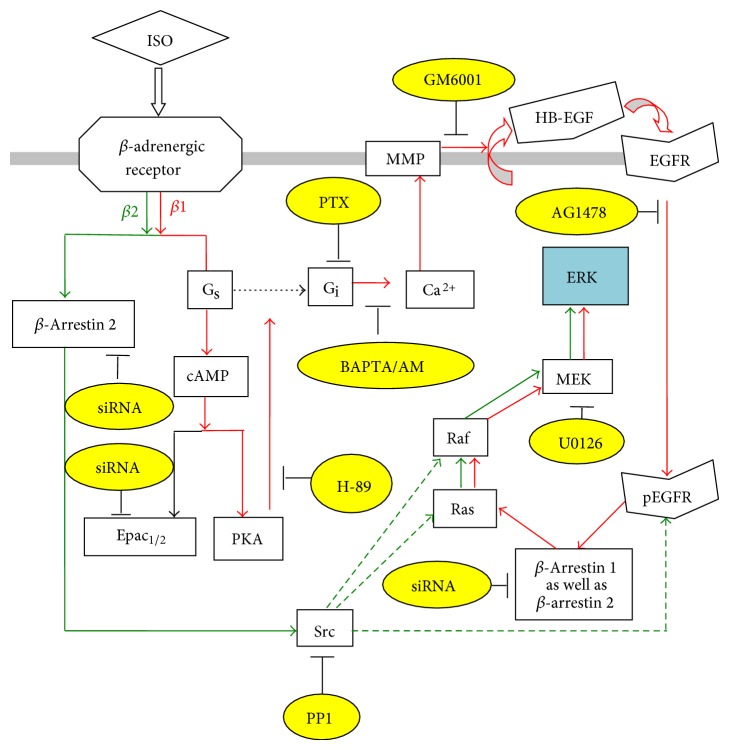
Schematic illustration of stimulation of ERK phosphorylation by *β*-adrenergic receptors in astrocytes. Isoproterenol (ISO) binds to *β*-adrenergic receptors. At high concentrations (>1 *μ*M), the activation of the receptors induces *β*_1_-adrenergic (red arrows), PKA-dependent “G_s_/G_i_ switching,” which in turn induces an enhancement of intracellular Ca^2+^ concentration by Ca^2+^ release from intracellular stores. The latter activates Zn-dependent metalloproteinases (MMPs) and leads to shedding of growth factor(s). The released epidermal growth factor (EGF) receptor ligand stimulates phosphorylation of the EGF receptor in the same and adjacent cells. The downstream target of the EGF receptor, extracellular regulated kinase (ERK), shown in blue, is phosphorylated via the Ras/Raf/MEK pathway. As shown in yellow ovals the ERK phosphorylation by isoproterenol at high concentration can be inhibited by H-89, an inhibitor of protein-kinase A (PKA); by PTX, an inhibitor of G_i_ protein; by BAPTA/AM, an intracellular Ca^2+^ chelator; by GM6001, an inhibitor of Zn-dependent metalloproteinases; by AG1478, an inhibitor of the receptor-tyrosine kinase of the EGF receptor; by siRNA against *β*-arrestin 1 and less completely by siRNA against *β*-arrestin 2; and by U0126, an inhibitor of MEK, which directly phosphorylates ERK. In contrast, at low concentration (100 nM) *β*_2_-adrenergic (green arrows) activation of the receptors activates Src via recruitment of *β*-arrestin 2. Src in turn stimulates ERK phosphorylation and phosphorylates EGF receptors at different sites than *β*_1_-adrenergic stimulation, without involvement of the receptor-tyrosine kinase. Its ERK_1/2_ phosphorylation is secondary to MEK activation, which may be induced by direct activation of Raf or Ras by Src. The ERK phosphorylation by isoproterenol at low concentration can be inhibited by siRNA against *β*-arrestin 2; by PP1, a Src inhibitor; and by the MEK inhibitor U0126. The effect of most of these inhibitors on MCAO-induced edema was investigated and tabulated in Table 3.

All experiments were carried out in accordance with the USA National Institute of Health Guide for the Care and Use of Laboratory Animals (NIH Publications number 80-23) revised 1978, and all experimental protocols were approved by the Institutional Animal Care and Use Committee of China Medical University. The experiments were carried out using rats, because it would have been very difficult to effectively perform MCAO in mice, used routinely for our cultures. In general, rats and mice are believed to react in rather similar manners to brain ischemia. Male Sprague-Dawley rats, weighing 280–320 g, were housed in cages on a 12 h light/dark cycle in a temperature-controlled (23–25°C) colony room with free access to food and water. Focal brain ischemia was induced unilaterally by occlusion of the right middle cerebral artery (MCAO) as previously described by Longa et al. [29] in animals anaesthetized with pentobarbital sodium. A 30 mm segment of 4-0 Ethilon monofilament was gently introduced from the common carotid artery into the internal carotid artery lumen until a slight resistance was felt. The filament had been coated with polylysine, and its tip was rounded by heating. The filament was withdrawn 3 hr after induction of ischemia. The animals were generally decapitated 8 hr after reperfusion, but in some cases (part of Figure 1) before reperfusion, and the brains were removed for measurement of brain edema. Intraventricular injection of drugs or saline (into the control animals) was made 15 minutes before the occlusion. The drugs were gently injected intraventricularly, dissolved in 5 *μ*L physiological saline, at the following concentrations: ethacrynic 50 mM; betaxolol 3 mM; ICI118551 10 *μ*M; H89 500 *μ*M; PTX 20 *μ*M; PP1 1 mM; AG1478 100 *μ*M; GM6001 1 mM; U0126 1 mM. With a brain weight in the rat of 1-2 g the intracerebral concentrations are probably at least 100 times smaller but dependent upon the distribution, which may vary between the individual drugs.

### 2.3. Determination of Edema

Wet weights (WW) were separately measured in right hemispheres (ischemia side) and left hemispheres (control side) after removal of cerebellum, pons, and olfactory bulbs. Dry weights (DW) were determined after 24 hr heating at 120°C as described by Bigdeli et al. [30], and percentage brain water content in each sample was calculated as [(WW − DW)/WW] × 100. The difference in brain water content between the ischemic and control sides indicates the magnitude of the induced edema.

### 2.4. Cell Cultures

Primary cultures of astrocytes were prepared from newborn male or female Swiss mice as previously described [31, 32]. The neopallia of the cerebral hemispheres were aseptically isolated, freed of meninges, dissociated by vortexing, filtered twice through nylon meshes, diluted in culture medium, and planted in 60 mm Falcon culture dishes. The culture medium [32] was Dulbecco's medium or a very slightly modified Dulbecco's medium [31], both containing 5.4 mM KCl, 1.8 mM CaCl_2_, 7.5 mM glucose, and 20% horse serum. The cultures were incubated at 37°C in a humidified atmosphere of CO_2_/air (5 : 95%). The medium was exchanged with fresh medium of similar composition on day 3 and subsequently every 3-4 days. At day 3, the serum concentration was reduced to 10%, and after the age of 2 weeks, 0.25 mM dibutyryl cyclic AMP (dBcAMP) was included in the medium. This compound increases intracellular cyclic AMP and promotes differentiation in astrocyte cultures derived from newborn brain. Recently the usefulness of astrocyte cultures has been authoritatively reviewed by Lange et al. [33]. Close similarities between our cultures and freshly isolated astrocytes in not only levels but also development of aralar protein and mRNA expression [34] and in gene changes introduced by chronic treatment with fluoxetine [35] support their validity as models of their* in vivo *counterparts. Moreover, stimulation of glycogenolysis by elevated extracellular K^+^, which is essential for its uptake into astrocytes [17], only occurs after treatment with dBcAMP [36].

### 2.5. Determination of Intracellular K^+^ Concentration

Determination of potential increase in intracellular K^+^ concentration from its control value by an increase in extracellular K^+^ concentration or by dobutamide was determined as by Xu et al. [17], using the K^+^-sensing fluorescent drug PBFI-AM. At the start of the experiment astrocytes cultured on coverslips coated with polylysine and placed within a Falcon Primaria culture dish were loaded with 10 *μ*M PBFI-AM with 0.2% Pluronic F-127 in phosphate-buffered saline (concentrations in mM: Na^+^ 141, K^+^ 5.4, Cl^−^ 146, PO_4_^3−^ 0.44, HCO_3_^−^ 4.0, Ca^2+^ 1.3, Mg^2+^ 1.3, SO_4_^2−^, and glucose 10) for 45 min at 37°C. After 2 times of wash with similar saline, the coverslip was superfused with saline solution with or without Ca^2+^ for 2 min, after which KCl was added. Subsequently the fluorescence intensity was recorded in each of ~20 individual cells for 7 min with 20 sec interval at excitation wavelengths of 340 (F340) and 380 (F380) nm. The ratio F340/F380 is an arbitrary measurement of the intracellular K^+^ concentration, and its potential changes during the measurement are an indication of K^+^ uptake rates. No calibration was made to obtain absolute values.

### 2.6. Ouabain Effect on K^+^ Uptake Rate in Media with and without Ca^2+^

Unidirectional net uptake of K^+^ was determined by exposing each culture for only 1.00 min (securing initial uptake rates) to serum-free culturing medium containing ^42^K at 37°C. In order to exclude homoexchange between added ^42^K and intracellular K^+^, the cultures had initially been depleted for intracellular K^+^ by a 10–15 min incubation in ice-cold (1°C) K^+^-free medium [37]. In order to rapidly reheat the cultures the 1 min incubation was carried out with the cultures floating on the surface of a water bath at 37°C. When ouabain was used it was present from the start of the preincubation.

After the 1 min incubation in the presence of ^42^K the cultures were washed three times with ice-cold 300 mM sucrose solution. In each wash all adherent fluid in the culture dish was removed with the aid of a fine nozzle connected to a water-suction pump. The whole washing procedure lasted for approximately 8 s. Such a short washing time is essential to prevent a significant decrease of ^42^K from the cells by passive efflux. The effectiveness of this washing procedure has previously been shown by very low amounts of potassium uptake at 1°C [31]. Subsequently, 1.00 mL of 1.0 M NaOH was added, ^42^K activity was counted in the cultures and in the incubation media with the aid of a Beckman FL800 gamma counter, and protein content was determined by the conventional Lowry technique [38].

Uptake rates were calculated from the uptake of ^42^K into the tissues and the specific activities of the media with due consideration of the rapid decay of ^42^K. The inhibition by ouabain was determined from the differences in uptake rates between cultures containing the inhibitor and cultures of the same batch from which it was absent. A tendency was observed towards a biphasic inhibition by ouabain, reflecting differences between its effect on the *α*_1_ and *α*_2_ subunit of the Na^+^,K^+^-ATPase.

### 2.7. Statistics

The statistical values of the differences between individual groups were analyzed by one-way ANOVA followed by Fisher's LSD test. The level of significance was set at *P* < 0.05. GraphPad Prism 6 analysis of the results shown in Figure 3 was performed assuming a biphasic inhibition.

## 3. Results

### 3.1. Edema Occurs after Reperfusion

Table 1 shows brain swelling in the ipsi- and contralateral hemispheres induced by MCAO. The determinations were made either immediately after the ischemic period (3 hr) or after the tissue had been reoxygenated for 8 hr. The results show that the edema appears after the reoxygenation with little, if any, edema formation during the ischemic period. This probably reflects insufficient energy to drive the Na^+^,K^+^-ATPase during the ischemic insult per se.

### 3.2. Effect of an NKCC1 Inhibitor or *β*_1_-Adrenergic Antagonists

The cotransporter inhibitor, ethacrynic acid, completely inhibited the effect of Na^+^,K^+^-ATPase/NKCC1 activation following transient ischemia/reperfusion, strongly suggesting that it was due to the increase in extracellular K^+^ concentration resulting from the ischemia (Table 2). The *β*_1_-adrenergic antagonist betaxolol also abolished edema formation, whereas the *β*_2_-adrenergic antagonist ICI118551 had no effect, identifying *β*_1_-adrenergic stimulation as the cause of the edema.

### 3.3. Effects of Inhibitors of *β*_1_- and *β*_2_-Adrenergic Signaling

The signaling pathways for both *β*_1_- and *β*_2_-adrenergic signaling H89 have been determined in cultured astrocytes with the aid of specific inhibitors [39]. As shown in Figure 1, phosphorylation of extracellular regulated kinase 1 and kinase 2 (ERK_1/2_) by isoproterenol is inhibited by H89, an inhibitor of protein-kinase A (PKA) and thus of G_s_-mediated signaling by isoproterenol. Pertussis toxin (PTX), an inhibitor of the G_s_-G_i_ switch occurring in the astrocytic signaling pathway of *β*_1_- but not *β*_2_-adrenergic signaling [39], has a similar effect. In this pathway G_s_ activation is followed by an increase in [Ca^2+^]_i_, which in astrocytes leads to metalloproteinase-mediated release of a growth factor that transactivates the epidermal growth factor receptor (EGFR). Both the metalloproteinase inhibitor GM6001 and the EGFR inhibitor AG1478 inhibit ERK_1/2_ phosphorylation. So does U0126, an inhibitor of ERK_1/2_ phosphorylation. As can be seen from Table 2 each of these inhibitors also prevents *β*_1_-adrenergic edema formation* in vivo* after ischemia/reperfusion. In contrast, the Src inhibitor PP1 that is active in the *β*_2_- but not *β*_1_-adrenergic signaling [39] had no effect. The consistency between results obtained in cultured astrocytes and the present* in vivo* results further supports the validity of the used cultures as models of their* in situ* counterparts. This is important for the studies described below.

### 3.4. Ca^2+^ Dependence of K^+^-Mediated Increase in Intracellular K^+^ Concentration

**Figure 2:**
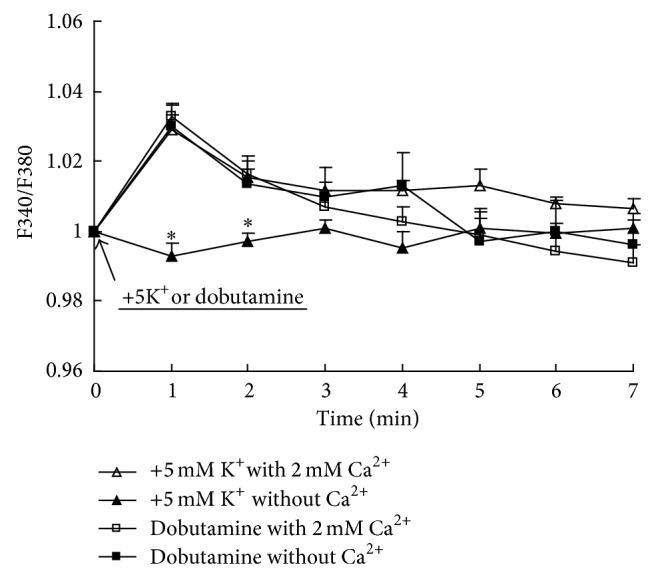
Uptake of K^+^ into primary cultures of astrocytes measured with the aid of the fluorescent drug PBFI-AM as described in Section 2. After incubation of PBFI-AM-loaded cells in saline solution for 45 min and subsequent wash, the cells were perfused either in similar solution with 2 mM CaCl_2_ or in a solution without CaCl_2_ for 2 min. From the start of the graphs they were perfused in a solution to which an additional 5 mM KCl or 10 *μ*M of the *β*_1_-adrenergic agonist dobutamine had been added at zero time (with a corresponding reduction of NaCl concentration when K^+^ was added). Results for K^+^ addition are averages from 60 individual cells on three coverslips and those after addition of dobutamine are from 38 cells. SEM values are indicated by vertical bars. ^*^Statistically significant (*P* < 0.05) difference from +5 mM K^+^ with 2 mM Ca^2+^ group at the same time period and from addition of dobutamine in either the presence or absence of Ca^2+^.

Figure 2 shows that in the presence of Ca^2+^ in the medium addition of 5 mM KCl causes a relatively fast increase in intracellular K^+^ concentration similar in magnitude to that previously observed [17]. In the absence of extracellular Ca^2+^ no such increase occurs. In contrast the effect of the *β*_1_-adrenergic agonist dobutamine is independent of the presence of Ca^2+^.

### 3.5. The Potency of Ouabain Is Reduced in the Absence of Extracellular Ca^2+^

**Figure 3:**
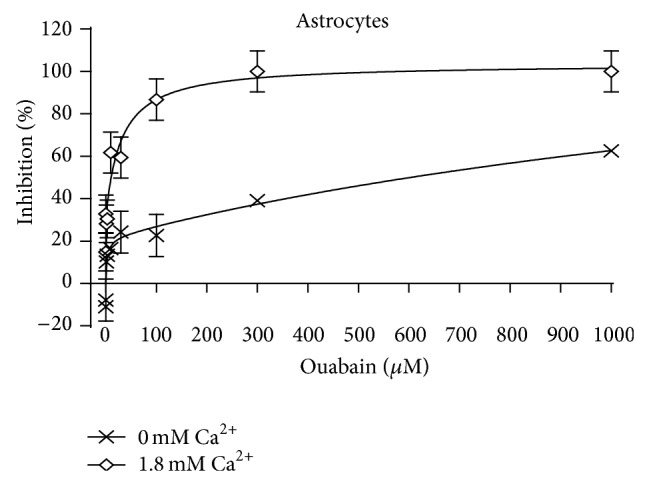
Ouabain induced inhibition of net influx of ^42^K into primary cultures of mouse astrocytes. The uptake was determined during a 1.00 min incubation, which provides initial uptake rates. The graph marked by diamonds was obtained in a slightly modified Dulbecco's medium containing 1.8 mM Ca^2+^ (for further details see Section 2) and that marked by X in a corresponding medium containing no CaCl_2_. The inhibition was calculated from the ratios between uptake rates in the absence of ouabain and that at the ouabain concentration in question. The calculated inhibitions were analyzed as a function of the ouabain concentration by GraphPad Prism 6, assuming that they are biphasic, which gave the best fit. SEM values for inhibition of cultures incubated at 1.8 mM Ca^2+^ were obtained from the standard errors given in the Graph Pad analysis for, respectively, high and low affinity. For cultures incubated without Ca^2+^ SEM value for the lower concentrations (high affinity) were similarly based on the Graph Pad analysis. A similar procedure could not be used for the low-affinity component, since the total inhibition did not approach 100%, and the computed standard error accordingly was excessively high. Therefore no SEM values are shown for the two highest concentrations, but they must resemble those at the lower concentrations, since the original uptake readings had approximately similar uncertainties at all concentrations. The apparent stimulation at low ouabain concentrations in the absence of Ca^2+^ reflects an ouabain-mediated stimulation of uptake, normally occurring at low nanomolar concentrations [17]. In both graphs 6 cultures from 2-3 different batches had been used for each point.

Figure 3 shows inhibition of ^42^K uptake by *μ*M concentrations of ouabain, expressed as percentage of the uninhibited rate in cultures incubated either in medium with a normal Ca^2+^ concentration (1.8 mM CaCl_2_) or in medium without added Ca^2+^. Since no Ca^2+^ chelator was added, this medium is likely to contain traces of Ca^2+^. The K^+^ concentration in the medium was 5.4 mM with ^42^K adding ~0.3 mM. Under control conditions (no ouabain present) the uptake rates were identical in the presence and absence of Ca^2+^ (275, resp., 282 nmol/min per mg protein). However, the potency of ouabain was drastically reduced by ouabain as seen from Figure 3. In both types of media the GraphPad analysis showed that a minor part of the uptake (~20%) is inhibited by the lower ouabain concentrations with high affinity, and a major part of the uptake is inhibited by higher ouabain concentrations. Unfortunately the individual measurements have been lost, preventing calculation of SEM values. However, some statistical information can be obtained from the GraphPad analysis, as discussed in the legend of the figure, and the statistical significance of the difference with and without Ca^2+^ is obvious at 100 *μ*M ouabain. The potency of ouabain on the fraction with lower affinity was always inhibited by Ca^2+^ deficiency. However, at the lowest ouabain concentrations there was a tendency towards a negative inhibition, that is, a stimulation, mimicking the stimulatory effect on Na^+^,K^+^-ATPase activity normally only observable at low nanomolar ouabain concentrations [17].

## 4. Discussion

### 4.1. Brain Edema and *β*_1_-Adrenergic Antagonists

This study has demonstrated that the ionic alterations evoked by ischemia (increase in extracellular K^+^ concentration, decrease in extracellular Ca^2+^ concentration) have profound effects on edema formation and its inhibition by *β*_1_-adrenergic antagonists. The finding that edema does not develop during the ischemic period but only after reoxygenation is consistent with the energy dependence of the Na^+^,K^+^-ATPase/NKCC1 transport system and indicates that the MCAO effectively prevented blood supply. It is in agreement with the observation by Goyagi et al. [40] that administration of *β*_1_-adrenergic antagonist 30 min after the onset of a 2-hour-long ischemic period drastically reduces infarct size and improves neurological deficit score after 7 days. Iwata et al. [27] even found that administration of antagonists specifically of the *β*_1_-adrenoceptor beginning 60 min after 8 min bilateral carotid artery occlusion combined with hypotension reduced neuronal injury after forebrain ischemia, although motor activity was not improved. Goyagi et al. [25, 26] also found less edema and infarct after administration of *β*_1_-adrenergic antagonist beginning before the insult, and motor deficit index scores were significantly lower and neuronal survival was better in rats treated with *β*_1_-adrenoceptor antagonists beginning 30 min before 10 min of spinal cord ischemia [28]. Unfortunately, the Goyagi group also found that although the *β*_1_-adrenergic antagonists provided long-term improvement of histological outcome, they had no effect on neurological outcome and spatial memory retention 14 days later, tested with administration beginning 30 min before the onset of ischemia and continuing for 24 hrs [26]. However, this does not necessarily indicate failure of *β*_1_-adrenergic antagonists to improve long-term functional recovery in brain ischemia. This is because *β*_1_-adrenergic stimulation exerts beneficial effects, such as the stimulation of glycogenolysis, which is essential for formation of memory [41–44]. It may therefore be advantageous to discontinue treatment with *β*_1_-adrenergic antagonist as soon as extracellular K^+^ concentration has normalized. This will in the presence of *β*_1_-adrenergic antagonist probably mainly be by neuronal uptake. It is unlikely to require ouabain-mediated signaling, since it in astrocytes serves to promote Na^+^ uptake for stimulation of the intracellular site of the Na^+^,K^+^-ATPase [17], whereas neurons increase intracellular Na^+^ during excitation.

### 4.2. NKCC1 Antagonists

The demonstrated ability of ethacrynic acid to abolish ischemia/reperfusion-induced swelling is consistent with its inhibition of swelling and uptake of Na^+^, K^+^, and Cl^−^ induced in monkey brain cortex slices by a highly elevated K^+^ concentration [7]. It is an indication that operation of NKCC1 is by far the dominant mechanism for removal of highly elevated extracellular K^+^ concentrations in brain. This is consistent with the conclusion by Walz and Hertz [45] that the increase in intracellular K^+^ content caused by elevation of extracellular K^+^ from 5 to 54 mM is entirely carrier mediated. A potential neuroprotective ability of ethacrynic acid has previously been studied in a combined trauma/hypoxia model of brain injury in the cat and in a small clinical trial [46]. Ethacrynic acid also had toxic side effects, which do not seem to be shared by furosemide and the very specific NKCC1 blocker bumetanide [47]. As already mentioned bumetanide inhibits edema in experimental stroke following middle cerebral occlusion (MCAO) and subsequent reperfusion [2–4]. In cultured astrocytes it diminishes cell swelling and Na^+^, K^+^, and Cl^−^ accumulation after oxygen and glucose deprivation [48] as well as trauma-induced astrocyte swelling [49].

### 4.3. Ca^2+^ and Ouabain Signaling

The rates for active K^+^ uptake in the absence of ouabain (~280 nmol/min per mg protein) are consistent with previously observed rates of K^+^ uptake in similar cultures [31] and the biphasic effect is explained by binding to two different Na^+^,K^+^-ATPase subunits [50]. The drastic decrease in ouabain potency in the absence of added Ca^2+^ in the medium (Figure 3) is in agreement with observation by Wang et al. [51] that a transmitter-induced rise in cytosolic Ca^2+^ triggers an increase in K^+^ uptake in cultured astrocytes, which was abolished by the NCX inhibitors SEA0400 and SN-6. The same astrocyte-specific transmitter also evoked a transient decrease in extracellular K^+^ in hippocampal slices. Finally, glucose utilization in freely behaving rats in the presence of elevated extracellular K^+^ concentrations [52] confirms a previously observed stimulation of oxygen consumption rate in brain slices [53, 54]. The threshold concentration of K^+^ evoking this effect (20 mM) [54] coincides with that causing swelling in brain slices [8] and the stimulation is inhibited by ethacrynic acid [55], indications that it is a metabolic manifestation of K^+^-stimulated K^+^ uptake. In the present context it is important that the K^+^-induced metabolic stimulation is abolished when Ca^2+^ is excluded from the medium [54].

Although the present observations together with literature data thus strongly suggest that extracellular Ca^2+^ depletion plays a major role by abolishing K^+^-induced, but not *β*_1_-adrenoceptor-mediated stimulation of the Na^+^-K^+^-ATPase/NKCC1 transport system, additional factors may also be involved in edema formation. Thus, the cellular increase in Ca^2+^, which is the reason for its extracellular decrease, promotes NADH hyperoxidation and electrical dysfunction after anoxia in hippocampal slices [56]. Not only neurons are affected by the Ca^2+^ overload, since cultured astrocytes rapidly die following anoxia and reperfusion when the gaseous and interstitial ionic changes of transient brain ischemia are simulated, and their death required external Ca^2+^ [57]. Importantly, brain swelling requires additional fluid uptake across the blood-brain barrier, which similarly expresses NKCC1 [58]. To what extent this may have played a role after the intraventricular injection of *β*_1_-adrenergic agonists (or of ethacrynic acid) used in the present study is unknown. Finally, it should be noted that prestroke use of *β*_1_-blockers in a thorough study was found not to affect stroke severity or outcome clinically [59]. This could, however, perhaps reflect that patients on continuous *β*_1_-blocker treatment might receive doses providing lower intracerebral concentrations than those employed in this study and needed for protection against edema. To counteract edema formation it may be essential to use high doses of *β*_1_-adrenergic antagonists until extracellular K^+^ has normalized. It is unknown how long that takes, but an at least hour-long increase in extracellular glucose level after seizures [60], which also increase extracellular K^+^ level, might suggest that this could take a long time, especially when astrocytic K^+^ reaccumulation is inhibited.

## 5. Conclusions

The beneficial effects of *β*_1_-adrenergic inhibition on infarct size and edema in experimental ischemia/reperfusion are convincing, even when added after the onset of ischemia. This is a great advantage for potential clinical use, and it is consistent with the present observation in this study that most or all of the swelling occurs after reperfusion. The study also confirmed an antiedema effect of NKKC1 inhibitors and of *β*_1_-adrenergic antagonists and signaling inhibitors, and it presented evidence that abnormal Ca^2+^ homeostasis resulting from ischemia is the pathophysiological basis for the mechanism by which *β*_1_-adrenergic inhibitors can inhibit brain edema after anoxia/reperfusion. This seems to be because Ca^2+^ deficiency counteracts stimulation of the Na^+^,K^+^-ATPase/NKCC1 transport system by elevated extracellular K^+^, which requires signaling by endogenous ouabains, and is thus dependent on extracellular Ca^2+^. Accordingly *β*_1_-adrenoceptor activity is left as its only astrocytic stimulus, and it was shown to be independent of the presence of extracellular Ca^2+^. Even in the presence of a *β*_1_-adrenergic antagonist extracellular K^+^ will eventually be reabsorbed by the neuronal Na^+^,K^+^-ATPase, which is unlikely to require extracellular Ca^2+^, but this will not lead to swelling in adult brain, where NKCC1 is highly enriched in astrocytes.

## Figures and Tables

**Table 1:** Brain water content in MCAO model with and without reperfusion.

	No reperfusion		8 hr reperfusion	
	Left hemisphere	Right hemisphere	Left hemisphere	Right hemisphere
Control	77.58 ± 0.20 (*n* = 5)	78.05 ± 0.29 (*n* = 5)	77.34 ± 0.18 (*n* = 3)	77.32 ± 0.14 (*n* = 3)
Ischemia 3 h	77.25 ± 0.16 (*n* = 5)	78.14 ± 0.25 (*n* = 5)	77.97 ± 0.17 (*n* = 8)	81.28 ± 0.34^*^ (*n* = 8)

Water content was calculated as [(wet weight − dry weight)/wet weight] × 100% in rats where MCAO had been performed on the right side. In control rats no significant change occurred with or without reperfusion. In animals with MCAO in the right hemisphere a small apparent increase in water content in this hemisphere after 3 hr of ischemia was not statistically significant, whereas a larger increase after reperfusion marked with ∗ was significant (*P* < 0.05). It was also significantly different (*P* < 0.05) from the small apparent increase without reperfusion.

**Table 2:** Brain water content in MCAO model after 3 hr ischemia and 8 hr reperfusion in the right hemisphere under control conditions (only saline injected intraventricularly before the occlusion) and after injection of a NKCC1 inhibitor or *β*_1_- or *β*_2_-adrenergic subtype-specific antagonists, dissolved in saline.

	Left hemisphere	Right hemisphere
Saline	77.97 ± 0.17 (*n* = 8)	81.28 ± 0.34 (*n* = 8)^*^
Ethacrynic	77.46 ± 0.11 (*n* = 7)	78.29 ± 0.76 (*n* = 7)
Betaxolol	78.02 ± 0.17 (*n* = 7)	78.44 ± 0.58 (*n* = 7)
ICI118551	77.78 ± 0.32 (*n* = 7)	81.08 ± 0.18 (*n* = 7)^*^

In rats with MCAO in the right hemisphere drugs were added 15 min before the occlusion as described in Methods. Water content was calculated as [(wet weight − dry weight)/wet weight] × 100%. In control animals (the same value as in Table 1) an increase in the ipsilateral hemisphere was significant (*P* < 0.05), as marked with ∗. In the presence of ethacrynic acid or the *β*_1_-adrenergic antagonist betaxolol no significant effect was seen, but in the presence of the *β*_2_-adrenergic antagonist ICI118551 water increased significantly (*P* < 0.05) in the ipsilateral hemisphere as marked with ∗.

**Table 3:** Brain water content in MCAO model after 3 hr ischemia and 8 hr reperfusion in the right hemisphere under control conditions (intracerebral saline only) and after injection of inhibitors of either the *β*_1_- or the *β*_2_-adrenergic pathway in astrocytes.

	Left hemisphere	Right hemisphere
Saline	77.97 ± 0.17 (*n* = 8)	81.28 ± 0.34 (*n* = 8)^*^
H89	77.00 ± 0.42 (*n* = 3)	77.19 ± 0.09 (*n* = 3)
PTX	77.19 ± 0.11 (*n* = 4)	77.51 ± 0.26 (*n* = 4)
GM6001	77.08 ± 0.11 (*n* = 4)	77.15 ± 0.13 (*n* = 4)
AG1478	77.14 ± 0.11 (*n* = 3)	77.27 ± 0.04 (*n* = 3)
U0126	77.39 ± 0.10 (*n* = 4)	78.22 ± 0.67 (*n* = 4)
PP1	77.52 ± 0.26 (*n* = 5)	80.04 ± 0.33 (*n* = 5)^*^

In rats with MCAO in the right hemisphere drugs were added 15 min before the occlusion as described in Methods. Water content was calculated as [(wet weight − dry weight)/wet weight] × 100%. In control animals (the same value as in Table 1) an increase in the ipsilateral hemisphere was significant (*P* < 0.05), as marked with ∗. This was also the case after treatment with PP1, an inhibitor of Src, an intermediate in *β*_2_-adrenergic signaling, but not after administration of any of the other inhibitors, which interrupt *β*_1_- but not *β*_2_-adrenergic signaling as shown and discussed in Figure 1. Most but not all inhibitors used to delineate the signaling pathways shown in that Figure were tested in this table. Since PP1 was the only *β*_2_-adrenergic inhibitor tested, an *n* = 5 was used, whereas each the other inhibitors, which all act on the same, *β*_1_-adrenergic were tested in fewer experiments.
